# Reintegration of the regenerated and the remaining tissues during joint regeneration in the newt *Cynops pyrrhogaster*

**DOI:** 10.1002/reg2.28

**Published:** 2015-04-08

**Authors:** Rio Tsutsumi, Takeshi Inoue, Shigehito Yamada, Kiyokazu Agata

**Affiliations:** ^1^ Department of BiophysicsGraduate School of ScienceKyoto UniversityKyotoJapan; ^2^ Human Health ScienceGraduate School of MedicineKyoto UniversityKyotoJapan; ^3^ Congenital Anomaly Research CenterGraduate School of MedicineKyoto UniversityKyotoJapan

**Keywords:** EFIC, joint, limb, newt, regeneration, reintegration

## Abstract

Urodele amphibians, such as newts, can regenerate a functional limb, including joints, after amputation at any level along the proximal−distal axis of the limb. The blastema can regenerate the limb morphology largely independently of the stump after proximal−distal identity has been established, but the remaining and regenerated tissues must be structurally reintegrated (matched in size and shape). Here we used newt joint regeneration as a model to investigate reintegration, because a functionally interlocking joint requires structural integration between its opposing skeletal elements. After forelimbs were amputated at the elbow joint, the joint was regenerated between the remaining and regenerated skeletal elements. The regenerated cartilage was thick around the amputated joint to make a reciprocally interlocking joint structure with the remaining bone. Furthermore, during regeneration, the extracellular matrix of the remaining tissues was lost, suggesting that the remaining tissues might contribute to the morphogenesis of regenerating cartilage. Our results showed that the area of the regenerated cartilage matched the area of the apposed remaining cartilage, thus contributing to formation of a functional structure.

## Introduction

Urodele amphibians, such as newts, have remarkable ability to regenerate a functional limb after amputation at any level along the proximal−distal axis of the limb (Bryant et al. 2002). After amputation, wound epidermis soon covers the wound surface, and the distal tip of the wound epidermis become a multilayered apical epithelial cap, which is necessary for blastema formation underneath it and is thought to correspond to the apical ectodermal ridge formed on a limb bud during development (Christensen & Tassava 2000; Christensen et al. 2002; Agata et al. 2007). After blastema formation, the molecular mechanisms of limb regeneration and limb development have much in common (Gardiner et al. 2002; Endo et al. 2004; Stoick‐Cooper et al. 2007; Nacu & Tanaka 2011).

If the forelimb blastema is transplanted to a different part of the body, such as a hindlimb stump or dorsal fin, the blastema can form a forelimb structure there (Pietsch 1961; Stocum 1968; Stocum & Melton 1977). Therefore it has been proposed that once proximal−distal identity has been established, the blastema can autonomously form the complete structure of the limb distal to the amputation site, including joints, without being affected by the stump.

At a joint, the opposing sides of the skeletal elements form a reciprocally interlocking structure. Such precisely reciprocal joint structures are essential for efficient and frictionless motion. Pathogenetically, malformation of the interlocking structure of the hip joint can cause osteoarthritis, with a complex sequence spanning embryonic, childhood, and adult life (Baker‐LePain & Lane 2010). Although little is known about how the convex−concave joint morphology is formed, the chondrogenesis and cell proliferation on opposing sides of the joint have been suggested to be involved in reciprocal joint morphogenesis (Pacifici et al. 2005). Furthermore, even when zeugopodal skeletal elements were abnormally formed in *Hox11* mutant mice, the joint between the normal stylopod and the abnormal zeugopod was remodeled and functionally organized, suggesting that the joint morphology is not pre‐patterned but rather is plastically determined by the interaction between the distal and proximal skeletal elements (Koyama et al. 2010).

**Figure 1:**
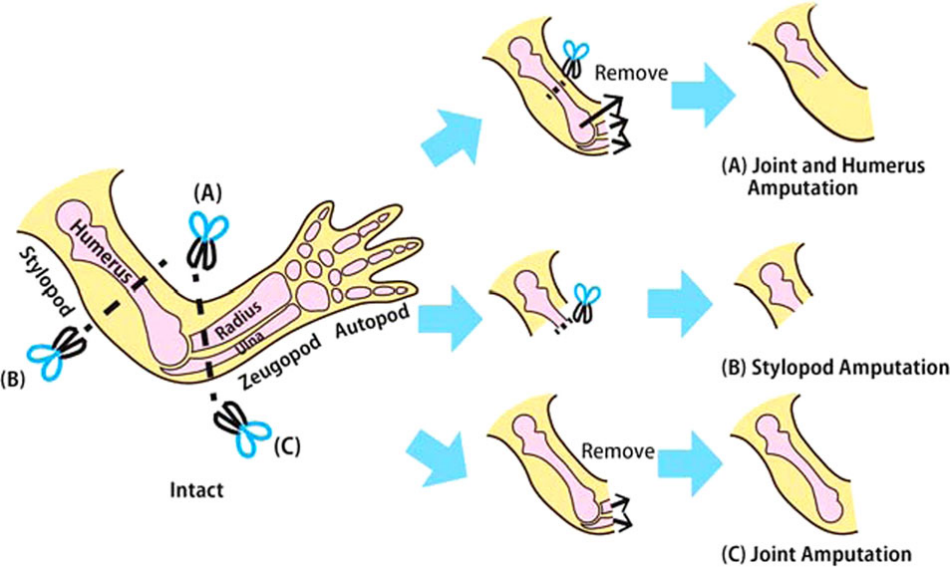
The methods of amputation. (A) For joint and humerus amputation, the forelimb was amputated slightly distal to the elbow joint and the residual amputated radius and ulna were removed, and then the humerus was amputated in its middle region. (B) For stylopod amputation, the forelimbs were amputated at the middle portion of the stylopod, and then the exposed humerus was trimmed. (C) For joint amputation, the forelimb was amputated slightly distal to the elbow joint, and then the amputated radius and ulna were removed.

Although the blastema can regenerate the limb morphology largely independently of the stump, the remaining tissues and the regenerated tissues must be structurally reintegrated to form a functional limb as a whole (Carlson 2007; McCusker & Gardiner 2014). Because a functional joint requires structural integration between the proximal and distal skeletal elements, we thought that regeneration of joints in the newt *Cynops pyrrhogaster* would provide a good model to clarify how the reintegration of the remaining tissues and the regenerated tissues occurs. For this purpose, here we compare the structure of the regenerated tissues after three different types of amputation. (1) The forelimb was amputated at the elbow joint, and then the humerus was amputated and removed, leaving the other stylopod tissues (such as skin, muscles, and tendons) intact (Fig. 1A). In this case, it would be expected that the bones of the zeugopod would be regenerated, while the bone of the stylopod would not be fully regenerated (Bischler 1926). (2) The forelimb was amputated at the stylopod (Fig. 1B). In this case, the bones of the stylopod and zeugopod would be regenerated. (3) The forelimb was amputated at the elbow (Fig. 1C). In this case, the bones of the zeugopod would be regenerated, while the bone of the stylopod would remain intact.

After the amputation, the remaining skeletal elements are ossified bone, whereas the regenerated skeletal elements are composed of cartilage. In order to observe the structure of the remaining and regenerated skeletal elements and to describe them quantitatively, it was necessary to obtain precise three‐dimensional (3D) images of both soft tissues and hard tissues. To achieve this, we used the technique of episcopic fluorescence image capturing (EFIC) (Weninger & Mohun 2002; Rosenthal et al. 2004; Tsuchiya & Yamada 2014). In EFIC, samples are embedded into a block and sectioned by an automatic microtome system. As the instrument is sectioning the block, every image of the tissues exposed in the block surface is captured with a microscope and charge‐coupled device (CCD) camera mounted on the microtome system by detecting the autofluorescence of the tissues. Finally, virtual 3D reconstruction is produced from the stack of these perfectly aligned and distortion‐free tissue images. In addition, the section slices can be collected and histologically stained to characterize the tissue type in detail. This method enabled us to quantitatively describe the morphological reintegration during newt forelimb regeneration. Our results shed some new light on the mechanism of tissue interaction for reintegration during amphibian limb regeneration.

## Results

### Confirmation of autonomous regeneration of the zeugopod and autopod independently of the remaining structure

**Figure 2:**
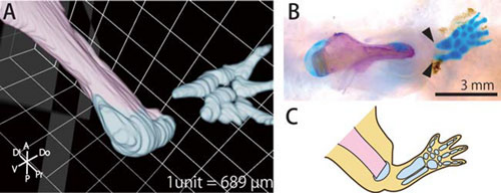
Morphology of the radius and ulna regenerated without interaction with the remaining humerus. (A) 3D image constructed using EFIC image of the regenerated skeletal elements after joint and humerus amputation. The remaining tissues are segmented in pink, and the regenerated tissues are segmented in blue. (B) Whole‐mount bone and cartilage staining of the regenerated skeletal elements after joint and humerus amputation. Bones are stained magenta, and cartilage is stained blue. The radius and ulna were regenerated without interacting with the remaining humerus, and in this case the proximal structures of the radius and ulna were not completely regenerated (arrowheads), as shown (C) in a schematic drawing.

In order to confirm whether the blastema can regenerate lost structures independently of the remaining tissues, we amputated newts’ forelimb at the elbow joint, and then removed the distal portion of the remaining humerus, including the elbow joint, without disturbing other remaining tissues such as muscles and tendons (Fig. 1A). As already reported (Bischler 1926), by 70 days after the amputation the blastema regenerated the zeugopod and autopod, while the humerus was not fully regenerated (Fig. 2A, B). The radius and ulna were regenerated without interacting with the joint of the humerus, strongly suggesting that the blastema can regenerate lost structures independently of the proximal remaining structures. However, the structures of the zeugopod skeletal elements were not completely regenerated. In particular, joint morphology was not regenerated in the proximal end of the zeugopodal skeletal elements; rather, the proximal end of the skeletal elements ended up with spike‐like morphology (Fig. 2), suggesting that the formation of the joint might depend on the interaction between the distal and proximal skeletal elements.

### Regeneration of functional elbow joint after amputation at the stylopod and the elbow

**Figure 3:**
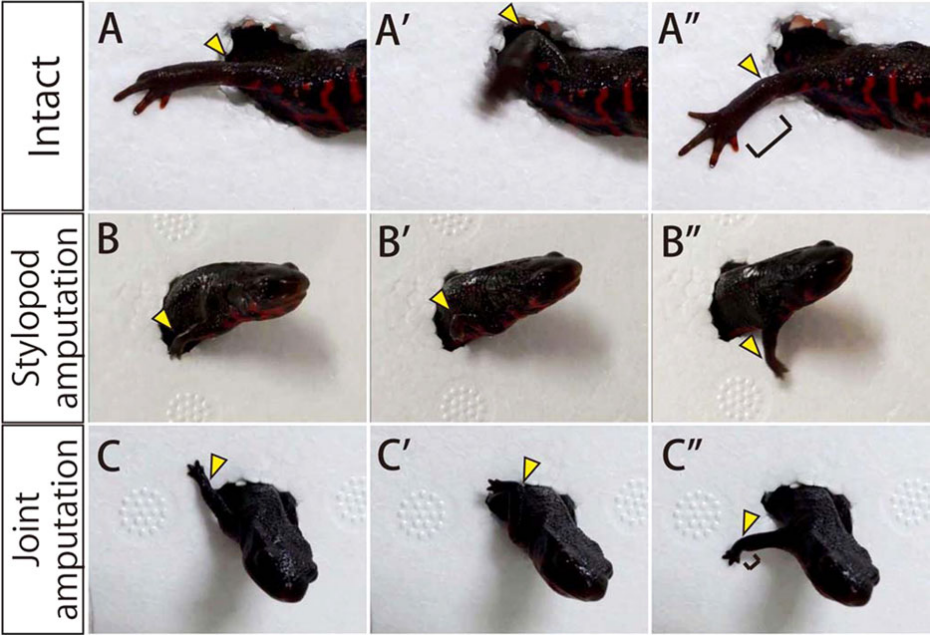
The motion of the regenerated joint after the amputation at upper arm and elbow joint (see also Movies S1−S3). (A, A′, A″) The bending−stretching motion of the intact elbow. The yellow arrowhead indicates the position of the elbow joint. (B, B′, B″) An equivalent bending−stretching motion of the elbow was also observed 70 days after stylopod amputation, and (C, C′, C″) 70 days after joint amputation. At 70 days after the amputation, the regenerated zeugopod and autopod were miniature sized compared with the intact zeugopod and autopod (square brackets).

To investigate whether the blastema can regenerate lost tissues without any interaction with the remaining tissues, we focused on regeneration of the elbow joint after the amputation of the forelimbs. Forelimbs were amputated at two different levels—at the middle portion of the humerus or at the elbow joint level, as shown in Fig. 1B and C—and the regeneration of the elbow joint was carefully observed 70 days after amputation. In order to evaluate whether a functional elbow could be regenerated, we observed the movement of the regenerated forelimbs as the newts passed through a short tunnel. As a control, intact newts showed bending−stretching motions of their elbow joint as they passed through the tunnel (Fig. 3A, A′, A″, yellow arrowheads; Movie S1). When the forelimb was amputated at the middle portion of the stylopod, the regenerated elbow joint showed smooth movement during such passage (Fig. 3B, B′, B″, yellow arrowheads; Movie S2), which was equivalent to that of the intact forelimb, suggesting that a functional elbow joint was regenerated by 70 days after amputation at the middle portion of the stylopod.

Regeneration of a functional elbow joint was also observed 70 days after the forelimb was amputated at the elbow joint level (Fig. 3C, C′, C″, yellow arrowheads; Movie S3). This suggested that the remaining stylopod and the regenerating zeugopod were reintegrated to regenerate a functional elbow joint between them, although the regenerating zeugopod and autopod were miniature sized (compare Fig. 3A″ with Fig. 3C″, square brackets).

### The regenerated cartilage might be reintegrated with the remaining tissues despite the discrepancy in size

**Figure 4:**
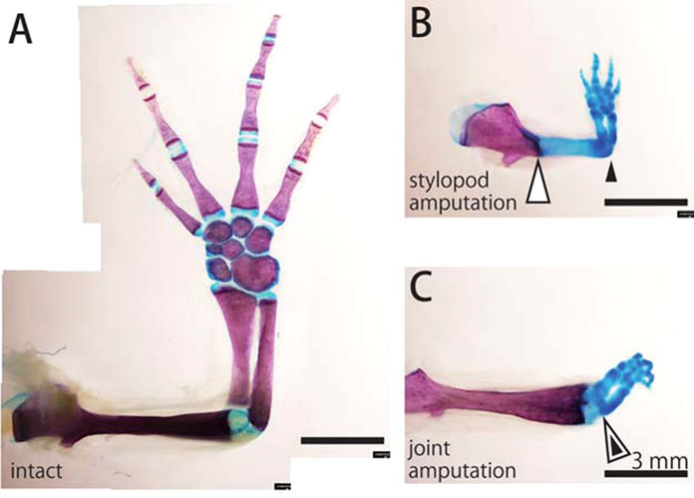
Skeletal structure of the regenerated limb. Whole‐mount bone and cartilage staining of (A) the intact forelimb, (B) the regenerated forelimb at 70 days after stylopod amputation, and (C) the regenerated forelimb at 70 days after joint amputation. Bones are stained magenta, and cartilage is stained blue. White arrowheads indicate the amputated site and black arrowheads indicate the regenerated elbow.

To observe the skeletal structure of the regenerated elbow joint, we performed alcian blue and alizarin red staining of the regenerated limbs. In the intact limb, the diaphysis of long bones and the cartilage were stained with alizarin red and alcian blue, respectively (Fig. 4A). At 70 days after the amputation in the middle of the stylopod, the regenerated structure was still miniature sized compared to the intact one (Fig. 4A, B). In this case, the elbow joint was regenerated between the miniature regenerated stylopod and the small regenerated zeugopod, and possibly for this reason there might have been no size discrepancy between the two opposite sides of the elbow joint. In contrast, at 70 days after amputation at the elbow joint, an elbow joint was regenerated between the large remaining stylopod and the miniature regenerated zeugopod despite their discrepancy in size (Fig. 4C), indicating that newts can regenerate a functional joint by reintegrating remaining tissues and regenerated tissues that have different sizes.

### 3D reconstruction and quantification of the shape of the remaining and the regenerated tissues

**Figure 5:**
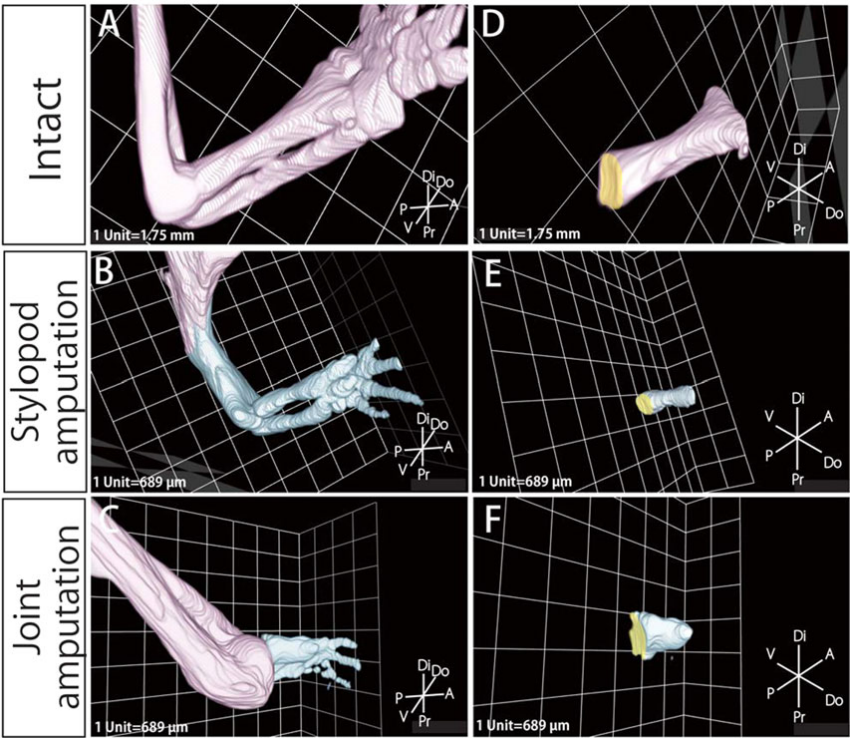
3D reconstruction image of bone and cartilage obtained by EFIC. The 3D reconstructed image of the skeletal elements in (A) the intact forelimb, (B) the regenerated forelimb at 70 days after stylopod amputation, and (C) the regenerated forelimb at 70 days after joint amputation. The remaining tissues are segmented in pink and the regenerated tissues are segmented in blue. We measured the volume of (D) the radius of the intact forelimb, (E) the radius at 70 days after stylopod amputation, and (F) the radius at 70 days after joint amputation. The joint surfaces of the radius whose areas were measured are segmented in yellow in (D), (E), and (F).

To examine how the remaining tissues and the regenerated tissues were reintegrated, we measured the sizes and quantitated the shapes of the remaining tissues and the regenerated tissues. Since the regenerated cartilage is a soft tissue whereas the residual intact bone is a hard tissue, we used EFIC to obtain a precise 3D image of the remaining bone and the regenerated cartilage (Fig. 5). In EFIC, images of the tissues at intervals along the *Z*‐axis were automatically captured, and then the remaining tissues was segmented in pink and the regenerated tissues in blue on every image (Fig. S1). From these images, the 3D reconstructed images of the intact forelimb (Fig. 5A) and of the regenerated limb at 70 days after amputation in the middle of the stylopod (Fig. 5B) and at 70 days after amputation at the elbow joint (Fig. 5C) were obtained. Using these images, we measured the volume and the area of the elbow joint surface of the radius of the intact forelimb, and of the regenerated limb at 70 days after the amputation in the middle of the stylopod and at 70 days after amputation at the elbow joint (yellow surface in Fig. 5D, E, F).

### The regenerated cartilage was thick near the boundary between the regenerated tissues and the remaining tissues

We speculated that the structure of the regenerated tissues around the amputated site was affected by the structure of the remaining tissues to facilitate their reintegration with each other, rather than that the regenerated structures were simply autonomously formed in the regenerated part. To begin to test this possibility, we decided to examine whether the regenerated structures that were far from the amputation site were autonomously developed and were approximately proportional to the intact ones. If the regenerated cartilage was almost proportional to the intact bone, the square root of the surface area of the corresponding parts should be proportional to the cube root of the volume.

**Figure 6:**
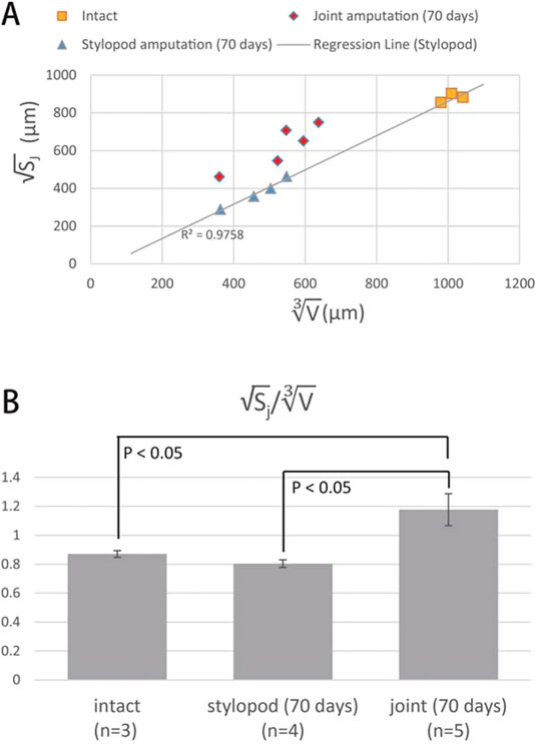
Morphological measurements of the regenerated joint. (A) Scattergraph of the cube root of the volume ($\sqrt[{3}]{V}$, *X* axis) plotted versus the square root of the joint surface area (√*S*_j_, *Y* axis) of the radius. The symbols represent the intact radius (orange squares), the regenerated radius 70 days after stylopod amputation (blue triangles), and the regenerated radius 70 days after joint amputation (red diamonds). The regression line was drawn for the regenerated radius after stylopod amputation. (B) The ratio between √*S*_j_ and $\sqrt[{3}]{V}$. The regenerated radius 70 days after joint amputation was significantly thicker around the joint compared with the intact radius and the regenerated radius after stylopod amputation (*P* < 0.05, Wilcoxon–Mann–Whitney test).

When we measured the radius of the regenerated stylopod at 70 days (Fig. 6A, blue triangles) after amputation in the middle of the stylopod, we found that the square root of the joint surface area (√*S*_j_) was proportional to the cube root of the volume ($\sqrt[{3}]{V}$) (*R* ^2^ = 0.976). Furthermore, by measuring the surface area of the joint and the volume of the intact radius, we found that the ratio between √*S*_j_ and $\sqrt[{3}]{V}$ of the intact radius (Fig. 6A, orange squares) was similar to that of the regenerated radius after amputation at the stylopod (*R* ^2^ = 0.997). This supports our hypothesis that the proportions of the regenerated radius are similar to those of the intact radius, and the shape of the regenerated radius is determined autonomously in the regenerated part.

In contrast, when we plotted the surface area versus the volume of the regenerated radius at 70 days after the amputation at the elbow (Fig. 6A, red diamonds), the values did not fall on the regression line corresponding to the regenerated radius after amputation in the middle of the stylopod, or that corresponding to the intact radius (Fig. 6A). Next, we calculated the ratio between the square root of the joint surface area and the cube root of the volume (√*S*_j_/$\sqrt[{3}]{V}$) (Fig. 6B). The results showed that the regenerated radius after the amputation at the elbow had a significantly higher ratio of √*S*_j_/$\sqrt[{3}]{V}$ compared to that after amputation in the middle of the stylopod and that in the intact elbow (*P* = 0.014 and 0.025, respectively, Wilcoxon−Mann–Whitney *U* test). This means that the regenerated radius was relatively thick at the joint, suggesting that the shape of the remaining tissues might affect the morphogenesis of the regenerated cartilage near the boundary between the regenerated and the remaining tissues.

### Some characteristics of the extracellular matrix in the remaining joint also change during regeneration after amputation at the elbow

**Figure 7:**
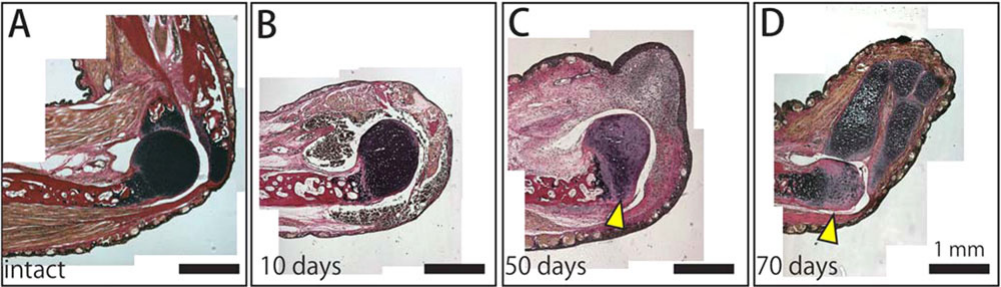
The process of regeneration after amputation at the elbow joint. Histological sections of the intact forelimb (A), and of the regenerated forelimbs at (B) 10 days, (C) 50 days, and (D) 70 days after joint amputation stained by Elastica van Gieson staining. Elastic fibers in cartilage are stained purple, collagen fibers in bones and tendons are stained red, muscles are stained yellow, and the cell nucleus is stained black. The signal for elastic fibers in the remaining joint was lost at 50 and 70 days after amputation (arrowheads).

To gain insight into how the regenerating tissues obtain information about the morphology of the remaining tissues, we performed Elastica van Gieson staining, which stains elastic fibers purple, collagen fibers in bone and tendon red, muscle fibers yellow, and the nucleus of cells black. Intact joints were strongly stained purple (Fig. 7A). Ten days after amputation at the elbow, when the wound epithelium completely covered the amputated site and before blastema formation, no significant change was observed for the staining in the remaining joint (Fig. 7B). In contrast, 50 days and 60 days after the amputation, when the blastema cells had started to differentiate and regenerated cartilage could be observed, respectively (Fig. 7C, D), the staining of elastic fibers had been lost in the remaining joint (Fig. 7C, D, arrowheads), suggesting that the tissue content of the remaining joint was altered during the regeneration process.

## Discussion

### Amputation at the elbow showed the importance of the reintegration system during joint regeneration

**Figure 8:**
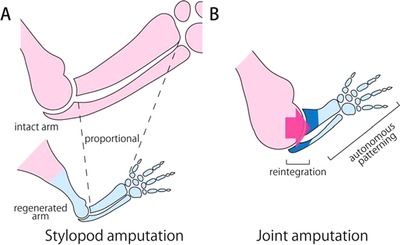
Schematic view of reintegration. (A) After stylopod amputation, the elbow joint is regenerated as a miniature of the intact elbow joint, such that the normal ratio between √*S*_j_ and $\sqrt[{3}]{V}$ of the radius is maintained, suggesting that the joint might be regenerated in a manner similar to the developmental and postnatal processes. (B) After joint amputation, the regenerated cartilage is thick around the elbow joint, suggesting that this regeneration occurs via mechanisms that depend on the size of the remaining humerus.

Our results showed that when the forelimb was amputated at the middle portion of the stylopod, the regenerated elbow joint was a miniature structure displaying the same proportions as the intact joint (Fig. 6A). A recent study revealed that the mechanisms of joint formation during regeneration are equivalent to those during development (Lee & Gardiner 2012). In development, the primordia of limb skeletal elements is first observed as a Y‐shaped uninterrupted mesenchymal condensation (Hinchliffe & Johnson 1980). The condensed mesenchymal cells at the future joint differentiate into interzone cells, whereas the other cells differentiate into chondrocytes (Holder 1977; Mitrovic 1978). As a result, the cartilaginous nodule is interrupted at future joint sites. The interzone cells differentiate into almost all joint tissues, such as synovial lining, ligaments, and articular cartilage (Koyama et al. 2008). The interlocking convex−concave structure of the joint requires reciprocal morphogenetic interactions, including chondrogenesis and cell proliferation of both the proximal and the distal skeletal elements (Pacifici et al. 2005). Thus, in the present study, when the elbow joint was regenerated from a relatively distant amputation site the elbow joint was reconstructed in a manner similar to the limb developmental process, and as a result the elbow joint grew bigger, as it does during postnatal limb growth, while maintaining the normal proportions of the regenerated skeletal elements (Fig. 8A).

In contrast, when the forelimb was amputated at the elbow joint with the humerus side of the elbow joint remaining intact, the elbow joint of the regenerated radius was larger than that of a proportionally miniature intact elbow (Fig. 6A, B). This result suggests that when the elbow joint is regenerated between the remaining and the regenerated skeletal elements, the morphology (including the size) of the regenerated skeletal elements reflects the morphology of the remaining joint (Fig. 8B). This supports the notion that there is a mechanism for reintegration of the remaining and the regenerating tissues, and in this mechanism the regenerating tissues might somehow obtain information about the morphology of the remaining tissues and use this information for proper reintegration. Furthermore, although the reintegration of the remaining and the regenerated tissues was clearly shown here just during joint regeneration after amputation at the elbow, we speculate that such reintegration also occurs in regeneration after amputation at any level along the limb (e.g. after amputation at the middle portion of the stylopod, the remaining proximal stylopod, and the regenerated distal stylopod). Thus, in this report, by examining regeneration after joint amputation, we have provided a quantitative description of morphological reintegration and suggest that this is a good model for analyzing the mechanism of reintegration.

### Blastema‐independent regenerative system might be responsible for reintegration

Regarding this matter, recently it has been proposed that the mechanism of limb regeneration can be divided into two different types of mechanism that are clearly distinguishable in accessory limb model (ALM) experiments: when the blastema is ectopically induced by nerve deviation, skin wounding, and skin graft, the limb skeletal elements are discontinuously regenerated on the soft tissues of the original limb (Endo et al. 2004); in contrast, when the original bone is wounded in addition to the regular ALM surgical procedure, the induced skeletal elements are integrated with the original bone (Satoh et al. 2010b). Thus, whereas the blastema epimorphically redevelops the distal structure, the tissues which are constructed by the blastema‐independent regenerative system connect the epimorphically regenerated tissues and the original tissues (Makanae et al. 2014). Such blastema‐independent regeneration can also be observed when a part of a skeletal element is excised inside the limb (Goss 1969; Hutchison et al. 2007; Satoh et al. 2010a). After such surgery, when the gap between the broken ends of the bone is smaller than a certain critical size, the gap is filled in with a skeletal outgrowth. Furthermore, when the excised area contains a joint, the joint can also be regenerated, indicating that the blastema‐independent regenerative system has the potential to regenerate the joint structure (Lee & Gardiner 2012). Therefore, the blastema‐independent regenerative system is thought to depend largely on proximal−distal tissue interaction, and this regenerative system might be responsible for the reintegration between the remaining part of an amputated joint and the regenerated part of the joint.

### What is the cellular and molecular mechanism of reintegration?

As shown by the loss of elastic fiber in the remaining joint of the humerus (Fig. 7), after amputation, the degradation and modification of the extracellular matrix (ECM) and dedifferentiation of the differentiated cells in the remaining stump tissues are observed accompanied by the expression of matrix metalloproteinases (Yang & Byant 1994; Yang et al. 1999; Vinarsky et al. 2005; Stevenson et al. 2006; Satoh et al. 2011). Furthermore, the positional information of blastema cells is labile so that they might acquire new positional information which is continuous to the proximal tissue (McCusker & Gardiner 2013). Although in principle blastema cells contribute to the limb tissues more distal to their origin, distal‐to‐proximal contribution has been reported at least in muscle cells (McCusker & Gardiner 2013; Nacu et al. 2013). Therefore, there is no sharp boundary between the blastema and stump tissues during regeneration; rather there should be an “intermediate zone” between the remaining part and the regenerated part and it might be important to focus on the event in the intermediate zone for understanding the mechanism of reintegration between the remaining and the regenerated part.

One possibility for the mechanism of reintegration is that the remaining tissues affect the amounts of the growth factors which stimulate the proliferation of dedifferentiated cells or those which recruit some cells. In the present study, the ECM of the remaining joint showed some changes during regeneration (Fig. 7). The ECM surrounding the chondrocytes in articular cartilage is responsible not only for mechanical properties of the cartilage but also for cellular properties of the chondrocytes as a result of effects on the binding of signaling molecules and transducing integrin‐mediated signals via ECM−cell interaction (García‐Carvajal et al. 2013; Loeser 2014). Therefore, during regeneration, changes in the ECM components of the remaining tissues might affect the expression and localization of signaling molecules in the remaining tissues, resulting in the regulation of cell proliferation, recruitment, and/or differentiation in the blastema. Actually, it has already been shown that the expression of certain genes was reactivated in the remaining tissues during newt jaw regeneration (Kurosaka et al. 2008).

An alternative possibility is that the distal end of the remaining humerus provides the regenerating joint with more cells after joint amputation than in the case in which the elbow joint is regenerated after stylopod amputation. In development, the joint cartilage and the central portion of the long bones are derived from distinct pools of chondrocytes (Koyama et al. 2008, Blitz et al. 2013; Sugimoto et al. 2013). However, we suppose that this possibility is not likely because, during regeneration, the cellular origin of regenerated cartilage is not strictly limited to the cartilage; rather the dermis can also contribute to cartilage via cell type conversion (Kragl et al. 2009).

It is also probable that the reintegrative morphogenesis of the regenerating joint requires physical contact with the remaining joint as a mold and mechanical force accompanied by bending−stretching motion. It has been shown in chicken and mouse that mobility during the embryonic stage is required for proper joint morphogenesis (Kahn et al. 2009; Roddy et al. 2011). Further studies using transplantation of the joint of the humerus, transgenesis, chimera analysis and genome editing techniques will reveal the molecular and cellular mechanisms of the reintegration of the remaining and regenerated tissues (Inoue et al. 2012; Hayashi et al. 2014).

### Future challenge for joint regeneration in non‐regenerative animals

One of the challenges in regenerative biology is to rescue the regenerative ability of non‐regenerative animals (Agata & Inoue 2012). Unlike the limb regenerative ability in urodele amphibians, that in mammals is limited to the digit tip (Muneoka et al. 2008). Some species of anuran amphibians, including *Xenopus*, have partial limb regenerative ability: after limb amputation, although a blastema is formed on the stump, they regenerate a hypomorphic single cartilaginous rod, called a spike (Goode 1967; Suzuki et al. 2006). Thus, *Xenopus* limb regeneration is regarded as a model of intermediate regenerative ability between those of urodele amphibians and mammals. During *Xenopus* spike formation, the limb blastema does not show the proper expression of genes essential for limb morphogenesis, such as the *Shh* and *Hox* genes, and possibly as a result the *Xenopus* limb blastema is not able to recapitulate the autonomous pattern formation system (Endo et al. 2000; Yakushiji et al. 2007; Ohgo et al. 2010). However, the findings in the present study suggest that the non‐autonomous pattern regeneration system may be responsible for joint morphogenesis, which may lead to a possible means of enhancing the regenerative ability of non‐regenerative animals.

## Materials and Methods

### Animals

Adult Japanese fire‐bellied newts, *Cynops pyrrhogaster*, were collected in Shiga prefecture, Japan. The animals were kept in plastic containers in filtered water at 19°C and fed once a week. All animals were maintained and manipulated according to a protocol approved by the Animal Care and Use Committee of Kyoto University.

### Forelimb amputation

Before surgery, animals were anesthetized by addition of 0.2% ethyl 3‐aminobenzoate methanesulfonate (Sigma‐Aldrich Co. LLC, St. Luis, MO) to their surrounding water for 10 min. For joint and humerus amputation, the forelimb was amputated slightly distal to the elbow joint and the residual amputated radius and ulna were removed with forceps. Then the muscle and connective tissues in the stylopod were peeled from the humerus, and the humerus was amputated at its middle region (Fig. 1A).

For stylopod amputation, the forelimb was amputated at the middle portion of the stylopod, and the humerus exposed due to the contraction of the amputated muscle was re‐amputated to flatten the amputated surface (Fig. 1B).

For joint amputation, the forelimb was amputated slightly distal to the elbow joint, and the amputated radius and ulna were removed with forceps in order to flatten the amputated surface and avoid destruction of the humerus (Fig. 1C). The regenerated forelimb was fixed overnight at room temperature with 4% paraformaldehyde, 10% methanol buffered in 70% phosphate buffered saline (PBS), and decalcified for 1–2 days with 22.5% formic acid, 10% sodium citrate, 70% PBS.

### EFIC Imaging

Sample preparation and EFIC imaging were performed as previously described, with some modifications (Weninger & Mohun 2002; Rosenthal et al. 2004; Yamada et al. 2010; Takaishi et al. 2014; Tsuchiya & Yamada 2014). Briefly, the fixed samples were dehydrated with ethanol and xylene and embedded in 70% paraffin wax, containing 25% Vyber, 4.4% stearic acid, and 0.4% Sudan IV. The blocks were sectioned at 10 μm thickness using a Leica SM2500 sliding microtome (Leica Microsystems, Wetzlar, Germany). The autofluorescence of tissues on the block surface was captured with a Hamamatsu ORCA‐ER low‐light CCD camera (Hamamatsu Photonics K.K., Shizuoka, Japan).

### 3D reconstruction and quantitative analysis

The tissues on 2D image stacks obtained by EFIC imaging were modified and segmented using Adobe Photoshop (Adobe Systems Inc., San Jose, CA). 2D image stacks were reconstructed into 3D images using Volocity (Improvision/Perkin Elmer, Waltham, MA).

For measurement of the surface area of the joint, the joint edge on each 2D image (resolution 5.12 μm/pixel) was traced with 5 pixel thickness using Adobe Photoshop ver. 13 (Adobe Systems), and 3D objects of the joint surface were reconstructed by stacking the traced edge. The volumes of the objects and of the shrunken objects which the software made by shrinking an object with 1 pixel thickness from each of the three axes were measured using Volocity (Improvision/Perkin Elmer). The surface area was calculated using the formula

$$S_{j}=(V-V_{s})/2RT$$

where *S*_j_ is the joint surface area, *V* is the volume of the object (μm^3^), *V*_s_ is the volume of the shrunken object (μm^3^), *R* is the resolution of the image (μm/pixel), and *T* is the thickness of shrinkage (1 pixel, in this case). The subtraction of the shrunken object from the object can be regarded as representing the superficial layer of the disk with 1 pixel thickness. Ignoring the side of the disk, the superficial layer of the disk represents two layers of the joint surface with 1 pixel thickness. Therefore, the surface area can be calculated by dividing the volume of these two layers by twice the thickness of the layers.

For measurement of the volume, the regenerated radius in each 2D image (the resolution was 5.12 μm/pixel) was segmented and the volume of the 3D reconstructed object was calculated using Volocity (Improvision/Perkin Elmer) as well.

### Elastica van Gieson staining

For histological analysis, the fixed samples were dehydrated with a series of increasing ethanol and xylene concentrations and embedded in paraffin wax. Tissue sections 10 μm thick were made, and the sections were stained with Elastica van Gieson staining. The images were obtained using an upright microscope BX62 (Olympus, Tokyo, Japan) and CCD camera CoolSNAP fx (Photometrics, Tucson, AZ).

### Whole‐mount bone and cartilage staining

For whole‐mount bone and cartilage staining, the samples were fixed with 70% ethanol for 1 h and 100% ethanol for 1 h. The cartilage was stained with 0.002% alcian blue in 20% acetic acid and 80% ethanol overnight, and washed with 100% ethanol. Tissues were decolorized with 0.5% KOH and 0.1% H_2_O_2_ for 3 h. The bones were stained with 0.01% alizarin red in 0.5% KOH for 6 h, and then the samples were cleared with glycerol. The images were obtained with a stereomicroscope Leica M125 (Leica Microsystems).

## Supporting information

Additional Supporting Information may be found in the online version of this article at the publisher's website:

**Movie S1**. The bending−stretching motion of the intact elbow.

**Movie S2**. The bending−stretching motion of the regenerated elbow after stylopod amputation.

**Movie S3**. The bending−stretching motion of the regenerated elbow after joint amputation.

**Figure S1**. Segmentation of EFIC image. (A) Raw image of EFIC. Autofluorescence of the tissues was detected. (B) Elastica van Gieson staining of sections obtained with EFIC. Sections were sliced with the microtome of EFIC and histologically stained to distinguish each tissue. (C) Segmentation of the tissues. By referring to the stained sections, the remaining tissues and the regenerated tissues in the image were segmented in pink and blue, respectively, and the joint surface was traced as a yellow line.
